# Health Economic Impact of Incomplete Reperfusion Patterns After Endovascular Thrombectomy in Acute Ischemic Stroke

**DOI:** 10.1007/s00062-025-01524-5

**Published:** 2025-08-28

**Authors:** Alexander Stebner, Petra Cimflova, Salome L. Bosshart, Wolfgang G. Kunz, Pervinder Bhogal, Michael Hill, Mayank Goyal, Johanna M. Ospel

**Affiliations:** 1 Department of Diagnostic Imaging, Foothills Medical Centre, University of Calgary, Calgary, Alberta Canada; 2 Department of Clinical Neurosciences, Foothills Medical Centre, University of Calgary, Calgary, Alberta Canada; 3 University of Basel, Division of Neuroradiology, Basel, Switzerland; 4 Universitätsinstitute für Diagnostische und Interventionelle Neuroradiologie, University Hospital Bern, Inselspital, Bern, Switzerland; 5 Department of Medical Imaging, St.Anne’s University Hospital Brno and Faculty of Medicine, Masaryk University Brno, Brno, Czech Republic; 6 University Hospital Basel, Department of Neurology, Basel, Switzerland; 7 Department of Radiology, University Hospital, LMU Munich, Munich, Germany; 8 Interventional Neuroradiology, Barts Health NHS Trust, London, UK; 9 University of Basel, Basel, Switzerland

**Keywords:** Stroke, Ischemia, Cost-effectiveness, Thrombectomy, Health economics

## Abstract

**Background and Purpose:**

Incomplete reperfusion in endovascular thrombectomy (EVT) impacts patients’ outcomes. Different incomplete reperfusion patterns may benefit from targeted therapeutic strategies, e.g. EVT-accessible incomplete reperfusion patterns could improve by performing additional EVT attempts, while EVT-non-accessible incomplete patterns might benefit from pharmacological therapies. The health-economic implications of these therapies are uncertain. This study aims to assess the potential economic benefits of improving incomplete reperfusion patterns after EVT.

**Materials and Methods:**

Retrospective Data analysis from the ESCAPE-NA1 trial, which included patients with large vessel occlusion strokes undergoing EVT. Reperfusion patterns were classified as near-/complete (eTICI 2c3), EVT-accessible incomplete (eTICI 2b), or EVT-non-accessible incomplete (eTICI 2b) and we compared multiple attempts to achieve eTICI 2c3 vs. first-pass eTICI 2c3. A Markov-Model was built to compare lifetime costs and quality adjusted life-years (QALY) for each reperfusion pattern over a lifetime horizon, considering both healthcare and societal perspectives.

**Results:**

A total of 1105 of patients were enrolled in the ESCAPE-NA1 trial of which 949 with eTICI 2b, 2c and 3 were further analyized (mean age 70.7 ± 13.6 [SD]; 463 female). Near-Complete reperfusion (eTICI 2c3) was achieved in 506/1105 patients (45.8%). Incomplete reperfusion patterns (eTICI 2b) were found in 450/1105 (40.7%) patients. Angiography imaging could be further investigated in 443/450 (98.4%) cases with 147/443(33.2%) EVT-accessible and 296/443(66.8%) EVT-non-accessible incomplete reperfusion patterns. Compared to EVT-accessible and EVT-non-accssible incomplete reperfusion, achieving complete (eTICI 2c3) reperfusion resulted in lower costs and an additional 1.14/0.45 QALYs, making it the dominant strategy from a health-economic perspective. In the complete reperfusion (eTICI 2c3) group, cumulative lifetime QALYs were similar with 5.25 for single-pass eTICI 2c3 and 5.19 for multi-pass eTICI 2c3.

**Conclusions:**

Improving incomplete reperfusion patterns after EVT has considerable potential health economic benefits, both in the presence and absence of a target occlusion that is amenable to EVT.

**Supplementary Information:**

The online version of this article (10.1007/s00062-025-01524-5) contains supplementary material, which is available to authorized users.

## Introduction

Over the past decade, endovascular thrombectomy (EVT) has revolutionized the treatment of acute ischemic stroke (AIS), as it is a highly effective treatment option [1], which not only improves the quality of life of stroke patients, but also reduces healthcare costs [2, 3].

Still, several factors can contribute to poor clinical outcomes after EVT and therefore also compromise its cost effectiveness. The time from symptom onset to treatment initiation [5], as well as the degree of reperfusion are crucially associated with outcomes in AIS [4, 5]. Despite recent improvements in EVT techniques, complete reperfusion is not achieved in all patients [6]. To rate the degree of reperfusion after EVT, the expanded Treatment In Cerebral Infarction (eTICI) score is used, ranging from 0 (no reperfusion) to 3 (complete reperfusion). An eTICI score of 2c or 3 is considered near-complete reperfusion which is the goal for EVT in AIS due to large vessel occlusion [7]. Although an eTICI score of 2b is considered “successful reperfusion”, it results in a 15–20% worse clinical outcome and also worse health economic outcomes compared to eTICI 2c3 [5, 8]. If eTICI 2b can be converted to eTICI 2c3, the final clinical outcome is comparable to those in whom near-complete reperfusion is immediately achieved [9].

Importantly, different “patterns” of successful, but incomplete reperfusion exist: in some patients, a visible target occlusion is present that may or may not be amenable to additional EVT attempts, while in others, only slow flow in distal vessels without a visible target occlusion is seen. Understanding the causes of these different patterns better could lead to development of different targeted strategies to improve reperfusion. A recent study has demonstrated that these different patterns of incomplete reperfusion have a significant impact on clinical outcomes [10]. Additionally, each additional EVT attempt might increase the risk of complications, such as vessel perforation and procedural complications, potentially leading to higher healthcare costs. However, studies have shown that, despite these risks, outcomes after multiple attempts to achieve eTICI 2c3 can be similar to those of first-pass success [9].

The health economic impact of these incomplete reperfusion patterns is currently uncertain. The objective of this study is to assess the cost-effectiveness of achieving complete (eTICI 2c3) reperfusion compared to different successful reperfusion (eTICI 2b) patterns, and to compare multiple attempts to achieve eTICI 2c3 vs. first-pass eTICI 2c3.

## Material and Methods

### Patient Sample

This is a secondary retrospective analysis of the prospective ESCAPE-NA1 trial. The ESCAPE-NA1 trial was a multicenter, randomized, double-blind, placebo-controlled study, that evaluated the effectiveness and safety of intravenous nerinetide, a neuroprotectant drug, in acute ischemic stroke patients with large vessel occlusion undergoing EVT. The study included 1105 patients across 48 acute care hospitals in eight countries. Ethics approval for the trial was obtained from the ethics committees at all participating centers, and consent was obtained from the patients or their surrogates according to local regulations. Detailed inclusion criteria have been published previously [11]. The decision regarding which EVT technique used was at the discretion of the operator, with the trial protocol imposing no specific rules or restrictions related to the device or technique.

### Angiographic Assessment—eTICI

The reperfusion status was determined by the ESCAPE-NA1 core lab. Incomplete reperfusion was defined as eTICI grade 2b, when antegrade reperfusion of more than half of the previously occluded target artery ischemic territory was reperfused and near-complete reperfusion was defined as eTICI 2c3, when either complete reperfusion was achieved (eTICI 3), or slow flow/occlusion in a few distal cortical vessels was visible such that at least 90% of the territory is perfused in the parenchymal phase or complete antergrade reperfusion of the previously occluded target artery ischemic territory is reperfused (eTICI 2c).

### Angiographic Assessment—Incomplete Reperfusion Patterns

**Fig. 1:**
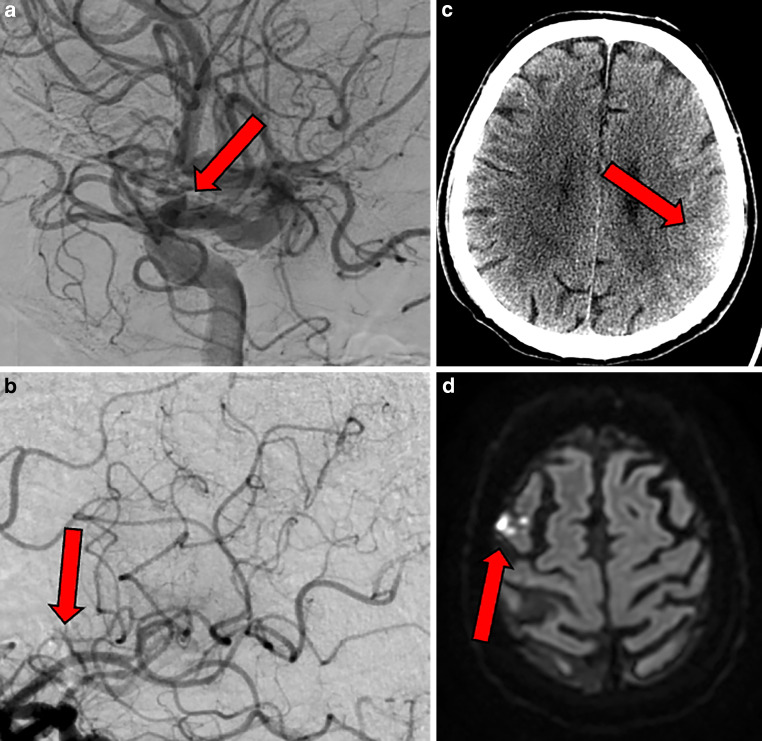
Illustrations of EVT-accessible and EVT-non-accessible occlusions. **a** shows a typical example of an EVT-accessible occlusion resulting in an eTICI 2b reperfusion. **b** shows the counterpart: a tiny, EVT-non-accessible occlusion potentially treatable with intra-arterial thrombolytics. **c** shows an example of how a potentially treatable, larger, and more proximal EVT-accessible occlusion can result in a larger, more uniform infarction. **d** shows tiny, scattered infarcts resulting from one or more smaller EVT-non-accessible occlusions

In cases with incomplete reperfusion (final eTICI 2b), presence or absence of residual occlusions was evaluated on the final intracranial angiography run with the first 50 cases read in consensus with an experienced neurointerventionalist (> 20 years of experience in diagnostics and neurointervention), the rest were read by a single expert reader (> 5 years of experience in stroke imaging and 1 year of training in neurointervention). EVT-accessible occlussions were defined as occulsions of the anterior or posterior M2 middle cerebral artery (MCA), proximal anterior or posterior M3 MCA or A2–A3 anterior cerebral artery (ACA) and if the vessel diameter was at least 1 mm and demonstrated a relatively straight course proximal to the occlusion as assessed on two dimensional digital subtraction angiography (DSA). EVT-non-accessible occlusion were any M3 MCA or A2–A3 ACA with diameter < 1 mm or markedly curved course proximal to the occlusion. Additional occlusion pattern were futher evaluated, detailed imaging acquisition criteria and analyses are described elsewere [10] and examples of EVT-accessible, as well as EVT-non-accessible occlusions are displayed in Fig. 1.

### Angiographic Assessment—First Pass vs. Multi-Pass eTICI 2c3

First pass eTICI 2c3 was defined as eTICI 2c or eTICI 3 reperfusion on the first EVT attempt. Multi-pass eTICI 2c3 was defined as eTICI 2c or eTICI 3 reperfusion that was achieved on the second or subsequent EVT attempts.

### Model Structure

We utilized TreeAge Pro 2022, version 2.0, to develop three separate decision models with two arms each, focusing on different scenarios of reperfusion in stroke patients. These models were built to assess costs and outcomes based on patients’ 30-month modified Rankin Scale (mRS) states over their lifetimes. Probabilities for functional outcomes were derived from the ESCAPE-NA1 trial and other relevant studies, while healthcare and societal costs were estimated using U.S. data, including costs for EVT and intravenous alteplase. The analysis considered both healthcare and societal perspectives. The incremental cost-effectiveness ratio (ICER) was used to compare cost-effectiveness between different reperfusion patterns, with upper and lower willingness-to-pay thresholds set at $100,000 and $ 50,000, respectively. The detailed model structure, as well as the base-case values and sources of the model input parameters can be found in the **supplementary material**.

### Model Probabilities

Probabilities of long-term outcomes were derived based on results of prospective cohort studies [12, 13] and the United States Life Tables [14], as outlined in prior studies [15–18]. These probabilities over the long term considered the risks of recurrent strokes, potential changes in the mRS after a stroke, and death. Utility weights obtained from a prospective cohort study were utilized to convert mRS states into quality adjusted life years (QALY) [19].

### Healthcare Costs

The U.S. National Inpatient Sample and current literature was used to estimated treatment costs (see **Supplementary Table 1**)[3, 15, 16, 20]). The total cost of EVT was estimated to be 15,510 USD, based on hospital charges and summary bills [3]. The estimated cost of intravenous alteplase in the US was 7421 USD [3, 21]. All expenses were adjusted for annual inflation based on the medical care component of the consumer price index [22].

### Societal Costs

The costs and effects of achieving near-complete reperfusion for EVT-accessible and EVT-non-accessible eTICI 2b patterns, as well as the costs of single versus multi-pass eTICI 2c3 patterns, were evaluated using the human capital approach, accounting for expenses associated with lost productivity (calculated based on data from the United States Bureau of Labor Statistics employment rates specific to age groups and probabilities of returning to work, adjusted for age and mRS scores [15, 16, 22]), informal care (uncompensated care provided by family and friends, calculated using wage data from the United States Census Bureau) and expenses arising from premature death with therefore loss of productivity, and disability related to stroke [16, 23].

### Analysis

**Fig. 2:**
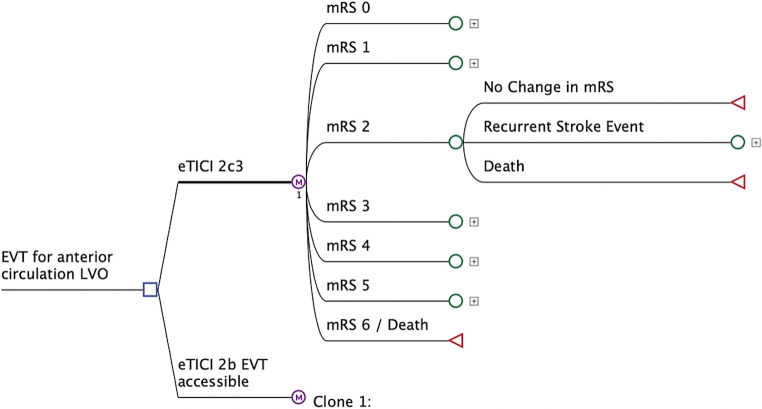
Cost-effectiveness model used in this analysis. The model has two arms, simulating lifetimes costs and quality adjusted life years for eTICI score of 2c/3 and eTICI 2b for EVT accessible patients. The model consists of an initial short-run model with a 3-month cycle and a long-run Markov state transition model with a 12-month cycle length. In the long-run component, patients in the mRS 0–5 health states could either remain in the same health state, suffer a recurrent stroke, and deteriorate to a worse health state or die, either due to recurrent stroke or age-related mortality. Purple round nodes indicate Markov (M) nodes, green round nodes indicate recursive nodes and red triangular nodes indicate terminal nodes

Figure 2 shows an example of the model structures for the analysis comparing eTICI 2c3 and EVT-accessible eTICI 2b reperfusion patterns. The same structure was used to compare eTICI 2c3 and EVT-non-accessible eTICI 2b patterns, and single-pass vs. multi-pass eTICI 2c3 patterns. In the base case analysis, the calculation of QALYs gained, lifetime costs, and the Incremental Cost-Effectiveness Ratio (ICER) relied solely on point estimates of model parameters as outlined in **Supplementary Table 1**, without considering the probability distributions for these parameters. Thereafter, in the probabilistic sensitivity analysis, a distribution was attributed to every model parameter and 10,000 second-order Monte Carlo simulations were performed for each set of analysis, enabling simultaneous fluctuation of all model parameters based on their respective distributions. The outcomes of the probabilistic sensitivity analyses were depicted in scatter plots. Additionally, we calculated health economic implications on a US population level, ie, for all patients in the US that receive thrombectomies in 2021 [24].

## Results

**Fig. 3:**
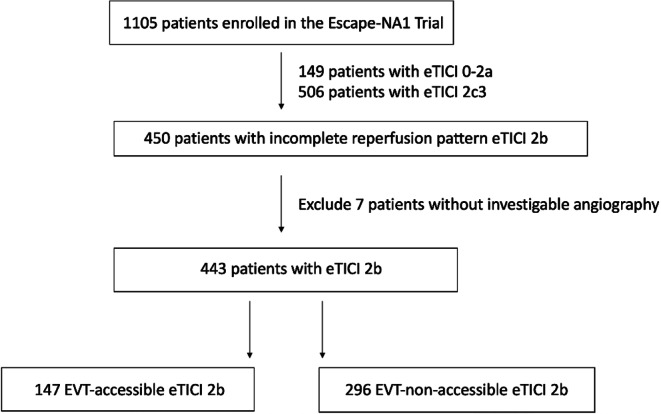
The flowchart shows inclusion and exclusion of patients. A total of 1105 patients were enrolled in the ESCAPE-NA1 trial. 450 patients had a final eTICI score of 2b. The angiogram of 7 patients were not sufficient for evaluation. The remaing 443 were devided into 147 EVT-accessible and 296 EVT-non-accessible eTICI 2b

In total 1105 patients participated in the ESCAPE-NA1 trial. Of these, 450 patients had a final eTICI score of 2b. In our analysis 7 patients had angiograms that could not be adequately assessed. The remaining 443 patients were categorized into 147 EVT-accessible and 296 EVT-non-accessible final eTICI 2b patterns. A flowchart of the inclusion and exclusion criteria is displayed in Fig. 3. The patients mean age was 70.7 ± 13.6. and 463 (48.8%) were female. Detailed patients characteristics are described in Table 1.

**Table 1:** Baseline characteristics of patients with eTICI 2c3, 2b-EVT-accessible and 2b-EVT-non-accessible

	Near complete reperfusion (eTICI 2c-3) *n* = 506	Incomplete reperfusion (eTICI 2b), MT-accessible pattern *n* = 147	Incomplete reperfusion (eTICI 2b), non-MT-accessible pattern *n* = 296	*p*-value
**Female sex—*n* (%)**	250 (49.4)	77 (52.4)	136 (46.0)	0.41
**Age—mean** **±** **SD**	70.2 ± 13.2	68.5 ± 13.8	68.1 ± 14.2	0.12
**Medical History—*n* (%)**				
Hypertension	370 (73.1)	96 (65.3)	200 (67.6)	0.09
Non-smoker (lifelong)	232 (46.1)	77 (52.4)	138 (46.8)	0.24
Hyperlipidemia	236 (46.6)	73 (49.7)	134 (45.3)	0.68
Atrial Fibrillation	194 (38.3)	56 (38.1)	85 (28.7)	0.02
Ischemic heart disease	117 (23.1)	35 (23.8)	71 (24.1)	0.95
Diabetes	99 (19.5)	27 (17.7)	66 (23.3)	0.56
Previous stroke or TIA	75 (14.8)	16 (10.9)	39 (13.2)	0.45
Chronic renal failure	31 (6.1)	9 (6.1)	18 (6.1)	1.00
Chronic heart failure	66 (13.0)	18 (12.2)	39 (13.2)	0.96
**Systolic blood pressure—mean** **±** **SD**	149 ± 25	147 ± 23	148 ± 25	0.74
**Baseline NIHSS—median (IQR)**	17 (13–21)	17 (11–21)	16 (12–20)	0.10
**Baseline ASPECTS—median (IQR)**	8 (7–9)	8 (7–9)	8 (7–9)	0.08
**Intravenous alteplase, yes—*n* (%)**	314 (62.1)	82 (55.8)	173 (58.5)	0.32
**Number of passes—median (IQR)**	1 (1–2)	2 (1–3)	1 (1–2)	< 0.001

Note: *ASPECTS* Alberta Stroke Program Early CT Score, *NIHSS* National Institutes of Health Stroke Scale, *TIA* transient ischemic attack

### Base Case Analysis

EVT with a final eTICI score of 2c3 resulted in 5.17 cumulative lifetime QALYs, and cumulative lifetime costs were 154,640 USD from a healthcare perspective and 185,800 USD from a societal perspective. An incomplete, EVT-accessible reperfusion patttern of eTICI 2b, in which the remaining occlusion was still considered amenable to further EVT attempts, resulted in 4.03 cumulative lifetime QALYs (difference of 1.14 QALYs). The lifetime costs from both the healthcare and societal perspectives were higher for EVT-accessible incomplete reperfusion patterns compared to complete reperfusion (160,828 USD healthcare perspective, 195,117 USD societal perspective). An EVT-non-accessible eTICI 2b pattern resulted in 4.73 cumulative lifetime QALYs (difference of 0.45 QALYs compared to the complete reperfusion pattern) and higher cumulative lifetime costs, namely 160,796 USD per patient from a healthcare perspective and 192,473 USD from a societal perspective.

Thus, in both groups, from a societal as well as from the healthcare perspective, complete reperfusion was the dominant strategy, resulting in lower cumulative lifetime cost and more cumulative lifetime QALYs (Table 2).

**Table 2:** Costs, QALY, ICER for EVT accessible 2b, non-accessible 2b and single vs. multi pass

	eTICI 2c/3	eTICI 2b, EVT accessible	Difference
**Cost-effectiveness of complete (eTICI 2c/3) vs. incomplete (eTICI 2b) reperfusion**			
Cumulative lifetime QALYs gained	5.17	4.03	1.14
Cumulative lifetime costs (healthcare perspective)—$	154,640	160,828	−6188
ICER (healthcare perspective)—$	EVT accessible 2b → 2c/3 dominant		
Cumulative lifetime costs (societal perspective)—$	185,800	195,117	−9317
ICER (societal perspective)—$	EVT accessible 2b → 2c/3 dominant		
–	*eTICI 2c/3*	*eTICI 2b, EVT non-accessible*	*Difference*
Cumulative lifetime QALYs gained	5.17	4.73	0.45
Cumulative lifetime costs (healthcare perspective)—$	154,640	160,796	−6156
ICER (healthcare perspective)—$	EVT not accessible 2b → 2c/3 dominant		
Cumulative lifetime costs (societal perspective)—$	185,800	192,473	−6473
ICER (societal perspective)—$	EVT not accessible 2b → 2c/3 dominant		
–	*Single pass*	*Multi pass*	*Difference*
**Cost-effectiveness of complete (eTICI 2c3) single vs. multi pass EVT**			
Cumulative lifetime QALYs gained	5.25	5.19	0.06
Cumulative lifetime costs (healthcare perspective)—$	154,407	149,680	4727
ICER (healthcare perspective)—$	90,458.94		
Cumulative lifetime costs (societal perspective)—$	186,010	179,797	6213
ICER (societal perspective)—$	118,907.28		

Note: *eTICI* expanded treatment in cerebral infarction, *QALY* Quality adjusted life years, *ICER* incremental cost-effectiveness ratio, *IVT* intravenous thrombolysis

Among the complete reperfusion (eTICI 2c3) group, cumulative lifetime QALYs were 5.25 for single-pass eTICI 2c3 vs. 5.19 for multi-pass patients eTICI 2c3 (difference 0.06). Cumulative lifetime costs from the healthcare (154,407 USD) and societal perspective (186,010 USD) were nominally slightly higher for single-pass eTICI 2c3 compared to multi-pass eTICI 2c3 (149,680 USD healthcare perspective, 179,797 USD societal perspective), although the magnitude of these differences was small.

### Probabilistic Sensitivity Analysis

**Fig. 4:**
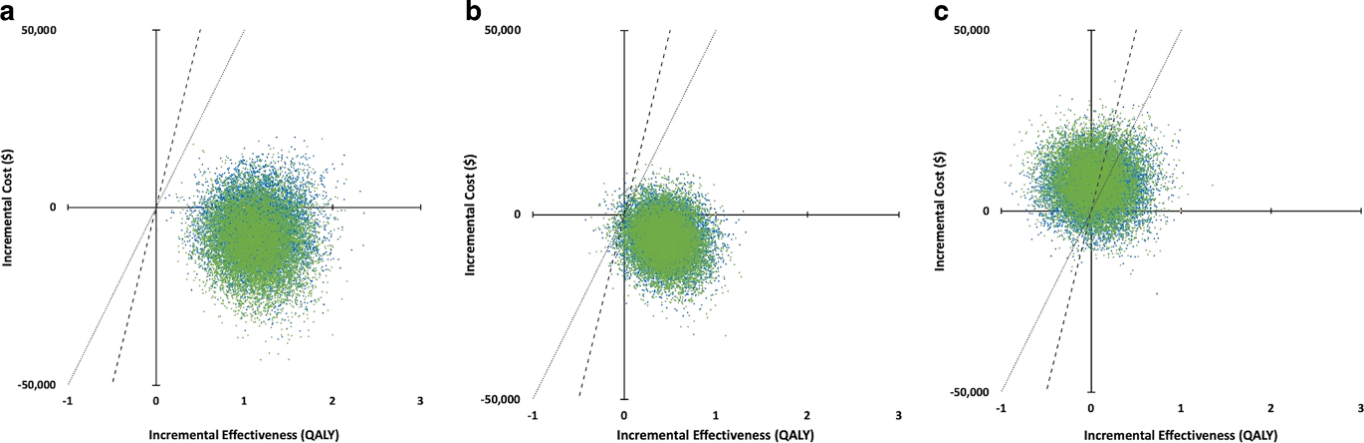
Probabilistic sensitivity analysis (10,000 Monte Carlo simulations) illustrating incremental costs (Y-axis) and incremental QALYs (X-axis) for large vessel occlusion stroke in the anterior circulation from a United States societal perspective (green dots) and healthcare perspective (blue dots). Each dot represents the result from a single Monte Carlo simulation. Dashed lines indicate 50,000/QALY willingness to pay thresholds, and dotted lines indicate 100,000/QALY willingness to pay thresholds. **a** shows incremental costs and QALYs for eTICI 2c3 vs. eTICI 2b, EVT-accessible. **b** shows incremental costs and QALYs for eTICI 2c3 vs. eTICI 2b, EVT-non-accessible. **c** shows incremental costs and QALYs for single-pass vs. multi-pass eTICI 2c3

The results of the probabilistic sensitivity analysis **(**Fig. 4**)** showed an acceptability of eTICI 2c3 vs. EVT-accessible eTICI 2b patterns of 100% from a healthcare and societal perspective for both the upper and the lower WTP thresholds. When compared to EVT-non-accessible eTICI 2b patterns, eTICI 2c3 patterns had an acceptability of 98.92%/99.11% for the healthcare and 98.65%/98.87% for societal perspective at the upper/lower WTP thresholds. For single- versus multi-pass eTICI 2c3 the acceptability from a healthcare perspective was 48.59%/37.94% at the upper and lower WTP thresholds and 45.31%/34.30% from the societal perspective.

### Implications On a US Population Level

In order to assess the health economic impact of incomplete reperfusion patterns on a population level, we calculated lifetime costs and QALYs for different reperfusion patterns on a US population level based estimated annual US EVT volumes published by Rai et al. [24]. This was based on an estimated number of 39,000 performed EVT procedures in the United States in the year 2019, and assuming incomplete reperfusion patterns in 53.9% based on proportions observed in the ESCAPE-NA1 trial (33.2% of which are EVT-accessible and 66.8% of which are EVT-non-accessible), improving the reperfusion rates in these patients to eTICI 2c3 would save 129 million USD (43 million USD for EVT-accessible, 86 million USD for EVT-non-accessible eTICI 2b patterns) from a healthcare perspective/156 million USD (65 million USD for EVT-accessible, 91 million USD for EVT-non-accessible eTICI 2b patterns) from a societal perspective annually, and result in 14,275 QALYs (7956 for EVT-accessible, 6319 for EVT-non-accessible eTICI 2b patterns) gained annually in the United States.

## Discussion

Our analysis suggests that there is a sizeable health economic benefit of improving reperfusion quality in patients with incomplete reperfusion patterns, both in patients who would be amenable to additional EVT attempts and in those in whom no EVT target occlusion is seen. Not surprisingly, compared to both these scenarios, achieving eTICI 2c3 resulted in lower lifetime costs and more lifetime QALYs, both from a healthcare and from a societal perspective, making complete (eTICI 2c3) reperfusion the dominant strategy. The potential health economic benefit of achieving complete reperfusion, both in terms of costs and QALYs, was larger in patients with EVT-accessible incomplete reperfusion patterns.

There is no doubt that EVT is nearly always cost-effective and in fact, often cost saving, in different healthcare settings and patient populations [15, 25, 26]. Furthermore, improved reperfusion quality has been shown to be closely associated with improved clinical and health economic outcomes outcomes [5]: clearly, achieving eTICI 2c3 vs. eTICI 2b results in better clinical outcomes, lower costs and more QALYs.

However, not all eTICI 2b cases are the same; and different “patterns” of incomplete reperfusion exist: in some cases, a clear residual occlusion is seen that is technically amenable to additional EVT attempts and could therefore potentially be recanalized. In other cases, incomplete reperfusion is caused by small, distal occlusions that are too peripheral to attempt EVT, or slow flow in distal vessels without any discernible occlusion. For the purpose of this analysis, we distinguished between EVT-accessible (ie, clear presence of a target occlusion that is amenable to EVT based on current expert opinion) and EVT-non-accessible patterns (ie, small, distal occlusions that are too peripheral to safely attempt EVT or slow flow without any visible occlusion).

Our results suggest that improving incomplete reperfusion patterns to complete recanalization is cost-effective regardless of the pattern of incomplete recanalization, but the potential health economic benefit is greater in patterns with EVT-accessible residual occlusions. This could be due to more severe strokes with bigger infarct volumes and therefore worse clinical outcomes in these patients, if no additional attempts to improve reperfusion quality are performed [10]. Furthermore, more proximal EVT-accessible occlusions might result in a more territorial infarct pattern, while small peripheral, non-EVT-accessible occlusions or slow flow in distal vessels may result more often in scattered infarct patterns, the latter of which has been shown to result in better clinical outcome than the former [27]. Lastly, in some instances, final eTICI 2c3 reperfusion may have been achieved by aggressive maneuvers that may have caused complications, and we were unable to asses the health economic impact of procedural complications in detail. That being said, severe complications would likely result in poorer functional outcomes at 90 days, and this would have been captured in our Markov model.

One critical question that remains unanswered is how we are going to improve reperfusion rates. While in patients with EVT-accessible occlusions, both mechanical (EVT) and pharmacological strategies (eg, intra-arterial thrombolysis, GPIIb/IIIa inhibitors) could be applied, patients with EVT-non-accessible occlusions only qualify for the latter. However, the optimal techniques and drugs that should be used, and their cost, remain unclear. Another question to answer is how far and how hard should neurointerventionalists push to achieve complete reperfusion, keeping in mind that additional EVT attempts increase procedural risk. A recent publication by Winkelmeier et. al. [28] showed no benefit of complete over incomplete reperfusion for patients with large ischemic strokes, who were systematically excluded in our dataset, and while this is in contradiction to most other studies, it still emphasizes that we should carefully compare the risks and benefits of additional mechanical reperfusion attempts.

For those patients with incomplete reperfusion who do not have EVT-accessible occlusions, pharmacological strategies would have to be used. Intraarterial Urokinase has already been shown to improve reperfusion after EVT [29] and could be one possible approach to turn eTICI 2b grades to eTICI 2c3. Ongoing studies like the TECNO-Trial [30], CHOICE II [31] and RE-ACT that investigate intraarterial thrombolysis treatment for residual occlusions will soon provide more data on the efficacy of such adjunctive pharmacological treatments.

Especially in our probabilistic analysis, we found only a small difference in health economic outcomes for single vs. multi-pass eTICI 2c3, with nominally improved outcomes in the multi-pass eTICI 2c3 group. This might reflect the fact that there was no clinically significant difference in our dataset when near-complete reperfusion was achieved with a single or multiple passes. This goes in line with the findings of Koge et. al. [9] showing no difference in clinical outcomes of patients in whom eTICI 2c3 was directly achieved, compared to those with multiple attempts. However, other data suggests that the so called first-pass effect, with achieving complete recanalization, directly, leads to better outcomes, which might translate into greater cost-effectiveness [32]. However, the results from our data in the ESCAPE NA1 sample showed no difference in terms of cost-effectivness between achieving eTICI 2c3 with a single or multiple attempts. Some of the studies describing improved health economic outcomes with first pass eTICI 2c3 used slightly older datasets, in which EVT devices that were used were potentially more traumatic than newer devices used in ESCAPE-NA1 [33]. As such, additional EVT attempts may have been potentially safer and less traumatic in ESCAPE-NA1 [34]. In conclusion, achieving eTICI 2c3 after multiple attempts using adjunctive mechanical and/or pharmacological stratgies is unlikely to compromise the cost-effectiveness of complete reperfusion. In fact, the improvement observed in multi-pass cases reinforces the value of persistent efforts to achieve near-complete reperfusion, even if multiple attempts are necessary.

## Limitations

First, there is some degree of inter-rater variability when applying the eTICI score [7], which may have introduced additional variability. Second, eTICI does not specify which areas were affected if reperfusion was incomplete, and as such, does not take into account brain eloquence. Third, the definition of EVT-accessible and non-accessible occlusions is somewhat subjective, based on expert judgement and rated by a single reader. It also depends on technical skills and is a moving target, as EVT tools and techniques continue to improve. Fourth, the eTICI score probably reflects a combination of recanalization and reperfusion, the latter of which is the more important factor that ultimately determines patient outcomes [35, 36]. Fifth, we have no clinical information on why the intervention was terminated at TICI 2b without proceeding to a higher degree of recanalization. These factors should ideally be better captured in future randomized clinical trials. In some cases this might have been necessary to avoid risky maneuvers, which could have led to higher recanalization rates but worse outcomes due to complications. Lastly, cost-effectiveness analyses are dependent on the quality of the underlaying data, which in our study were mostly derived from randomized controlled trials and prospective studies, and we used a US healthcare perspective for our analysis. The results are therefore expected to differ in other healthcare infrastructures, regions and countries [37].

## Conclusion

Improving incomplete reperfusion patterns after EVT has considerable potential health economic benefits, both in the presence and absence of a target occlusion that is amenable to EVT. More research is needed to develop and improve adjunctive EVT treatments aimed at improving reperfusion quality.

## Supplementary Information

Supplemental Methods and Results

## Abbreviations

ACA: anterior cerebral artery
AIS: acute ischemic stroke
eTICI: exlanded thrombolysis in cerebral infarction
EVT: endovascular thrombectomy
ICER: incremental cost-effectiveness ratio
IVT: intravenous thrombolysis
MCA: middle cerebral artery
mRS: modified Rankin scale
QALY: Quality-adjusted life-year
WTP: willingness to pay
