# Supplementary material for: Health Economic Impact of Incomplete Reperfusion Patterns After Endovascular Thrombectomy in Acute Ischemic Stroke
Source: Clin Neuroradiol. 2025 Aug 28;36(1):93–102. doi: 10.1007/s00062-025-01524-5 (PMC13009091; doi:10.1007/s00062-025-01524-5)
Supplement: Supplementary file 1 — Supplemental Methods and Results [file 62_2025_1524_MOESM1_ESM.pdf]

SUPPLEMENTAL MATERIAL

Model structure:

TreeAge Pro 2022, version 2.0 (TreeAge Pro Software, Inc.) was used to build three separate decision models with 2 arms. (1) The first arm of the first model represents patients with successfully achieved near-complete to complete reperfusion (eTICI 2c3) and the second arm contains patients with a reperfusion score of eTICI 2b with EVT-accessible occlusion that could potentially be converted into eTICI 2c3. (2) The second model has a similar structure with the first arm representing eTICI 2c3 patients and the second arm representing patients with a reperfusion score of eTICI 2b that is EVT-non-accessible (see definition in the patient sample selection section[1]). (3) The third model represents patients in whom complete reperfusion (eTICI 2c3) is achieved in the first pass in its first arm vs. after multiple passes in its second arm. In all three models, patients are assigned to one out of 7 health states represented by the modified Rankin Scale (mRS) that ranges from 0 (no disabilities) to 6 (death) in an initial 3-month cycle. Probabilities of these health states were based on the 90-day mRS distribution of patients in the respective reperfusion subgroups in the ESCAPE-NA1 trial. Following this initial short, single cycle, a long-run Markov state transition model with a cycle length of 1-year was used to estimate costs and outcomes from 3 months after the stroke over the patients' entire lifespan (up to 120 years). In the Markov model, patients could either maintain the same health status, suffer a recurrent stroke followed by either complete recovery to the same mRS or deterioration to a worse mRS. Age-related mortality (mRS 6) is also accounted for in the model based on data from US Life Tables. We assumed a model starting age of 70.9 years, matching the median age of in the ESCAPE-NA1 sample. All analyses in this study were conducted from two perspectives, as recommended by the Second Panel on Cost-Effectiveness in Health and Medicine[2] and the Consolidated Health Economic Evaluation Reporting Standards 2022 (CHEERS 2022) guidelines (checklist in the supplement)[3]: the healthcare perspective, which only takes into account healthcare-related costs, and the societal perspective, which also considers costs to society outside the healthcare sector, such as costs due to lost productivity, informal caregiving and premature mortality.

Model probabilities:

Probabilities of long-term outcomes were derived based on results of prospective cohort studies[4, 5] and the United States Life Tables[6], as outlined in prior studies[7-10]. These probabilities over the long term considered the risks of recurrent strokes, potential changes in the mRS after a stroke, and death. Utility weights obtained from a prospective cohort study were utilized to convert mRS states into quality adjusted life years (QALY)[11]. In the ESCAPE-NA1 trial, the probability of achieving an eTICI 2c/3 score was 46.1% (506/1105 patients) and incomplete final reperfusion (eTICI 2b) was found in 450 patients (41.0%)[12]. Angiography imaging was complete and further characteristics could thus be investigated in 443/450 (98.4%) patients, out of which 147 (33.2%) had EVT-accessible and 296 (66.8%) had EVT-non-accessible incomplete reperfusion patterns[1]. Input parameters for our models were derived from these patient subgroups. To investigate patients with single-pass vs. multi-pass eTICI 2c3, data from the ESCAPE-NA1 trial as described by Cimflova et al.[13] were used. Detailed probabilities for patient subgroups with the respective reperfusion patterns are displayed in **Supplementary Table 1.**

Healthcare Costs

The U.S. National Inpatient Sample and current literature was used to estimated treatment costs (see **Supplementary Table 1**)[7, 8, 14, 15]. The total cost of EVT was estimated to be 15,510 USD, based on hospital charges and summary bills[15]. The estimated cost of intravenous alteplase in the US was 7,421 USD[15, 16]. All expenses were adjusted for annual inflation based on the medical care component of the consumer price index[17].

Societal Costs:

The costs and effects of achieving near-complete reperfusion for EVT-accessible and EVT-non-accessible eTICI 2b patterns, as well as the costs of single versus multi-pass eTICI 2c3 patterns, were evaluated using the human capital approach, accounting for expenses associated with lost productivity (calculated based on data from the United States Bureau of Labor Statistics employment rates specific to age groups and probabilities of returning to work, adjusted for age and mRS scores[7, 8, 17]), informal care (uncompensated care provided by family and friends, calculated using wage data from the United States Census Bureau) and expenses arising from premature death with therefore loss of productivity, and disability related to stroke[8, 18].

Outcomes of interest:

Quality adjusted life years (QALY) were used to measure effectiveness, and costs were measured in US dollars ($)[2, 19]. QALYs for individuals in each mRS category were determined by multiplying life years by utility measures specific to the mRS. An annual discount rate of 3% was applied to both costs and QALYs[2, 11]. Cost-effectiveness of eTICI 2c3 vs. eTICI 2b patterns was assessed using the incremental cost-effectiveness ratio (ICER) which was calculated as follows:

$$\frac{(\text{Cost of eTICI 2c3}) - (\text{Cost of eTICI 2b pattern})}{(\text{QALYs of eTICI 2c3}) - (\text{QALYs of eTICI 2b pattern})}$$

$$\frac{(\text{Cost of singlepass eTICI 2c3}) - (\text{Cost of multipass eTICI 2c3})}{(\text{QALYs of singlepass eTICI 2c3}) - (\text{QALYs of multipass eTICI 2c3})}$$

Upper and lower willingness-to-pay (WTP) thresholds were set at 100,000 USD and 50,000 USD. We further calculated the mean net monetary benefit (NMB) and acceptability with respective 95% prediction intervals from probabilistic sensitivity analyses (10,000 second order Monte Carlo simulations). The NMB equals the product of the QALYs gained and the willingness to pay for one QALY minus the associated healthcare or societal costs.

*Net monetary benefit = (QALYs gained * willingness to pay for 1 QALY) – lifetime costs.*

Acceptability is the percentage of all Monte Carlo simulations in the probabilistic sensitivity analysis that show cost effectiveness, ie, that show an ICER below the willingness-to-pay threshold.

Supplementary table 1: Base-Case Values and Sources of the Model Input Parameters

Parameter	Expected value	Distribution	Reference
Probabilities for eTICI 2c3 pattern			
Probabilities for achieving mRS 0/ 1/ 2/ 3/ 4/ 5/ 6 – eTICI 2c3	21 / 27 / 21 / 11 / 5 / 5 / 10	Dirichlet	Hill et al.[12]
Probability for receiving IVT	0.621	Beta	Hill et al.[12]
Probabilities for eTICI 2b non EVT-accessible pattern			
Probabilities for achieving mRS 0/ 1/ 2/ 3/ 4/ 5/ 6	17 / 25 / 22 / 10 / 6 / 9 / 11	Dirichlet	Cimflova et al.[1]
Probability for receiving IVT	0.585	Beta	Cimflova et al.[1]
Probabilities for eTICI 2b EVT accessible pattern			
Probabilities for achieving mRS 0/ 1/ 2/ 3/ 4/ 5/ 6	14 / 17 / 19 / 16 / 8 / 10 / 16	Dirichlet	Cimflova et al.[1]
Probability for receiving IVT	0.558	Beta	Cimflova et al.[1]
Probabilities for single vs. multi pass eTICI 2c3 pattern			
Probabilities for achieving mRS 0/ 1/ 2/ 3/ 4/ 5/ 6 single pass	23 / 27 / 19 / 13 / 4 / 6 / 9	Dirichlet	Cimflova et al.[13]
Probability for receiving IVT in single pass EVT	0.612	Beta	Cimflova et al.[13]
Probabilities for achieving mRS 0/ 1/ 2/ 3/ 4/ 5/ 6 multi pass	18 / 28 / 26 / 8 / 6 / 2 / 11	Dirichlet	Cimflova et al.[13]
Probability for receiving IVT in multi pass EVT	0.629	Beta	Cimflova et al.[13]
Transition probabilities			
Recurrent stroke rate	0.059 (for first year)	Beta	Pennlert et al.[5]
Annual death rate	0.022 (for 71 years)	Beta	Arias et al.[6]
Annual death hazard rate ratios for mRS 0/ 1/ 2/ 3/ 4/ 5	1.53/ 1.52/ 2.17/ 3.18/ 4.55/ 6.55	Log-normal	Hong et al.[4]
After recurrent stroke	HERMES meta-analysis control arm	Dirichlet	Goyal et al.[20]
Healthcare costs			
Costs within first 90 days after stroke for mRS 0/ 1/ 2/ 3/ 4/ 5/ 6	$27,543/ $24,467 /$13,029/ $69,344/ $41,783/ $85,198/ $14,447	Gamma	Sevick et al.[21]

Additional cost of EVT	$17,834	Gamma	Shireman et al.[15]
Long-term annual costs after stroke for mRS 0/ 1/ 2/ 3/ 4/ 5	$12,458/ $12,828/ $14,840/ $25,482/ $51,575/ $75,825	Gamma	Shireman et al.[15]
Costs for hospitalization due to recurrent stroke	$26,972	Gamma	Gloede et al.[22]
Societal costs			
Median annual salary of employed population	$45,000 (for 72 years)	Gamma	US Census Bureau 2022
Population employment rate	0.258 (for 65-74 years)	Beta	US Bureau of Labor Statistics 2022
Relative earnings of stroke survivors	0.825	Beta	Vyas et al.[23]
Return to work probability after stroke for mRS 0/ 1/ 2/ 3/ 4/ 5	0.63/ 0.72/ 0.49/ 0.19/ 0.14/ 0.00	Beta	Tanaka et al.[24]
Informal annual caregiving costs	mRS 0–2: $5,261, mRS 3–5: $28,778	Gamma	Barral et al.[25]
Utilities mRS 0/ 1/ 2/ 3/ 4/ 5/ 6	1.00/ 0.91/ 0.76/ 0.65/ 0.33/ 0.00/ 0.00	Beta	Chaisinanunkul et al.[11]

Note: eTICI= expanded treatment in cerebral infarction, mRS = modified Rankin Score, EVT = endovascular treatment, IVT=intravenous thrombolysis.

Supplementary table 2: Mean net monetary benefits with respective 95% prediction intervals and acceptability for EVT accessible eTICI 2b, non-accessible eTICI 2b and single versus multi pass eTICI 2c3:

	**eTICI 2c3**	**eTICI 2b EVT-accessible**
Complete (eTICI 2c/3) vs. incomplete (eTICI 2b) recanalization		
Mean NMB (95%PI) at the upper/ lower WTP in $ - healthcare perspective	363,467 (362,890 – 364,044) / 104.390 (104.685 – 104.094)	243,086 (242,450 – 243,722) / 40.876 (41.224 – 40.528)
Acceptability of EVT at the upper/lower WTP - healthcare perspective	100% / 100%	
Mean NMB (95%PI) at the upper/ lower WTP in $ - societal perspective	332,206 (331,637 – 332,776) / 72.966 (73.268 – 72.664)	208,703 (208,071 – 209,335) / 6.845 (7.203 – 6.487)
Acceptability of EVT at the upper/lower WTP - societal perspective	100% / 100%	
	**eTICI 2c3**	**eTICI 2b EVT non-accessible**
Mean NMB (95%PI) at the upper/ lower WTP in $ - healthcare perspective	363,477 (362,905 – 364,050) / 104.390 (104.685 – 104.094)	312,217 (311,620 – 312,620) / 75.693 (76.015 – 75.372)
Acceptability of EVT at the upper/lower WTP - healthcare perspective	98.92% / 99.11%	
Mean NMB (95%PI) at the upper/lower WTP in $ - societal perspective	332,480 (331,912 – 333,048) / 73.135 (73.435 – 72.836)	281,083 (280,485 – 281,682) / 44.116 (44.444 – 43.787)
Acceptability of EVT at the upper/lower WTP - societal perspective	98,65% / 98,87%	
	**Single pass eTICI 2c3**	**Multi-pass eTICI 2c3**
eTICI 2c3 single vs. multi-pass EVT		
Mean NMB (95%PI) at the upper/ lower WTP in $ - healthcare perspective	368,620 (368,020 – 369,220) / 105.593 (105.903-105.283)	369,524 (368,845 – 370,203) / 109.863 (110.209-109.517)
Acceptability of EVT at the upper/lower WTP - healthcare perspective	48.59 / 37.94	
Mean NMB (95%PI) at the upper/ lower WTP in $ - societal perspective	336,821 (336,217 – 337,425) / 74.024 (74.341 – 73.708)	339, 871 (339,188 – 340,553) / 79.811 (80.163 – 79.459)
Acceptability of EVT at the upper/lower WTP - societal perspective	45.31 / 34.30%	

Note: NMB=net monetary benefit, WTP=willingness to pay threshold, eTICI= expanded treatment in cerebral infarction, upper WTP 100,000, lower WTP 50,000.

References:

1. Cimflova P, Singh N, Kappelhof M, et al. Effect of incomplete reperfusion patterns on clinical outcome: insights from the ESCAPE-NA1 trial. *J Neurointerv Surg* 2023 20230725. DOI: 10.1136/jnis-2023-020553.
2. Sanders GD, Neumann PJ, Basu A, et al. Recommendations for Conduct, Methodological Practices, and Reporting of Cost-effectiveness Analyses: Second Panel on Cost-Effectiveness in Health and Medicine. *Jama* 2016; 316: 1093-1103. DOI: 10.1001/jama.2016.12195.
3. Husereau D, Drummond M, Augustovski F, et al. Consolidated Health Economic Evaluation Reporting Standards 2022 (CHEERS 2022) Statement: Updated Reporting Guidance for Health Economic Evaluations. *Value Health* 2022; 25: 3-9. DOI: 10.1016/j.jval.2021.11.1351.
4. Hong KS and Saver JL. Years of disability-adjusted life gained as a result of thrombolytic therapy for acute ischemic stroke. *Stroke* 2010; 41: 471-477. 20100204. DOI: 10.1161/strokeaha.109.571083.
5. Pennlert J, Eriksson M, Carlberg B and Wiklund PG. Long-term risk and predictors of recurrent stroke beyond the acute phase. *Stroke* 2014; 45: 1839-1841. 20140501. DOI: 10.1161/strokeaha.114.005060.
6. Arias E, Heron M and Xu J. United States Life Tables, 2014. *Natl Vital Stat Rep* 2017; 66: 1-64.
7. Kunz WG, Hunink MG, Dimitriadis K, et al. Cost-effectiveness of Endovascular Therapy for Acute Ischemic Stroke: A Systematic Review of the Impact of Patient Age. *Radiology* 2018; 288: 518-526. 20180612. DOI: 10.1148/radiol.2018172886.
8. Sarraj A, Pizzo E, Lobotesis K, et al. Endovascular thrombectomy in patients with large core ischemic stroke: a cost-effectiveness analysis from the SELECT study. *J Neurointerv Surg* 2021; 13: 875-882. 20201113. DOI: 10.1136/neurintsurg-2020-016766.
9. Ospel J, Zerna C, Harrison E, et al. Cost-effectiveness of late endovascular thrombectomy vs. best medical management in a clinical trial setting and real-world setting. *Can J Neurol Sci* 2024: 1-18. 20240226. DOI: 10.1017/cjn.2024.19.
10. Ospel JM, Kunz WG, McDonough RV, et al. Cost-effectiveness of Endovascular Treatment for Acute Stroke with Large Infarct: A United States Perspective. *Radiology* 2023; 309: e223320. DOI: 10.1148/radiol.223320.
11. Chaisinanunkul N, Adeoye O, Lewis RJ, et al. Adopting a Patient-Centered Approach to Primary Outcome Analysis of Acute Stroke Trials Using a Utility-Weighted Modified Rankin Scale. *Stroke* 2015; 46: 2238-2243. 20150702. DOI: 10.1161/strokeaha.114.008547.
12. Hill MD, Goyal M, Menon BK, et al. Efficacy and safety of nerinetide for the treatment of acute ischaemic stroke (ESCAPE-NA1): a multicentre, double-blind, randomised controlled trial. *The Lancet* 2020; 395: 878-887. DOI: 10.1016/S0140-6736(20)30258-0.
13. Cimflova P, Ospel JM, Singh N, et al. Effects of reperfusion grade and reperfusion strategy on the clinical outcome: Insights from ESCAPE-NA1 trial. *Interv Neuroradiol* 2024: 15910199241288874. 20241014. DOI: 10.1177/15910199241288874.
14. AHRQ. HCUPnet: a tool for identifying, tracking, and analyzing national hospital statistics. [Available from: https://hcupnet.ahrq.gov/#setup.
15. Shireman TI, Wang K, Saver JL, et al. Cost-Effectiveness of Solitaire Stent Retriever Thrombectomy for Acute Ischemic Stroke: Results From the SWIFT-PRIME Trial (Solitaire

With the Intention for Thrombectomy as Primary Endovascular Treatment for Acute Ischemic Stroke). *Stroke* 2017; 48: 379-387. 20161227. DOI: 10.1161/strokeaha.116.014735.
16. Dawson J, Lees JS, Chang TP, et al. Association between disability measures and healthcare costs after initial treatment for acute stroke. *Stroke* 2007; 38: 1893-1898. 20070419. DOI: 10.1161/strokeaha.106.472381.
17. BLS. US Bureau of Labor Statistics: Consumer Price Index (CPI). https://www.bls.gov/cpi/ (accessed July 24 2021).
18. CPS. U.S. Census Bureau. Current Population Survey (CPS) - CPS Table Creator, https://www.census.gov/cps/data/cpstablecreator.html (accessed July 24 2021).
19. Prieto L and Sacristán JA. Problems and solutions in calculating quality-adjusted life years (QALYs). *Health Qual Life Outcomes* 2003; 1: 80. 20031219. DOI: 10.1186/1477-7525-1-80.
20. Goyal M, Menon BK, van Zwam WH, et al. Endovascular thrombectomy after large-vessel ischaemic stroke: a meta-analysis of individual patient data from five randomised trials. *Lancet* 2016; 387: 1723-1731. 20160218. DOI: 10.1016/s0140-6736(16)00163-x.
21. Sevick LK, Demchuk AM, Shuaib A, et al. A Prospective Economic Evaluation of Rapid Endovascular Therapy for Acute Ischemic Stroke. *Can J Neurol Sci* 2021; 48: 791-798. 20210112. DOI: 10.1017/cjn.2021.4.
22. Gloede TD, Halbach SM, Thrift AG, et al. Long-term costs of stroke using 10-year longitudinal data from the North East Melbourne Stroke Incidence Study. *Stroke* 2014; 45: 3389-3394. DOI: 10.1161/strokeaha.114.006200.
23. Vyas MV, Hackam DG, Silver FL, et al. Lost Productivity in Stroke Survivors: An Econometrics Analysis. *Neuroepidemiology* 2016; 47: 164-170. 20161220. DOI: 10.1159/000454730.
24. Tanaka H, Toyonaga T and Hashimoto H. Functional and occupational characteristics predictive of a return to work within 18 months after stroke in Japan: implications for rehabilitation. *Int Arch Occup Environ Health* 2014; 87: 445-453. 20130516. DOI: 10.1007/s00420-013-0883-8.
25. Barral M, Rabier H, Termoz A, et al. Patients' productivity losses and informal care costs related to ischemic stroke: a French population-based study. *Eur J Neurol* 2021; 28: 548-557. 20201106. DOI: 10.1111/ene.14585.
